# Bitter Taste Sensitivity, Food Cravings, and Risk of Chronic Disease: A Cross-Sectional Study

**DOI:** 10.7759/cureus.74509

**Published:** 2024-11-26

**Authors:** Tony Jehi, Hildemar Dos Santos, Gigi Kwok-Hinsley

**Affiliations:** 1 School of Public Health and Health Sciences, California State University, Dominguez Hills (CSUDH), Carson, USA; 2 Preventive Care, School of Public Health, Loma Linda University, Loma Linda, USA; 3 School of Public Health, Loma Linda University, Loma Linda, USA

**Keywords:** bitter taste, chronic disease risk, food cravings, tas2r38, taste sensitivity

## Abstract

Introduction: Variation in common taste receptor type 2 member 38 (TAS2R38) haplotypes is associated with bitter taste sensitivity, but there is not much or inconsistent evidence on association with food cravings and with chronic disease risk factors. We have conducted a cross-sectional study to assess whether genetically defined taster groups would differ in their sensitivity to bitter-tasting compounds, cravings for various food groups, and risk of chronic disease risk factors.

Methodology: A total of 116 non-diabetic individuals were recruited from the Loma Linda University (LLU) campus. Various measures were carried out to examine genetic variations, bitter sensitivity, anthropometrics, food cravings, and participant demographics. Taster status was determined based on TAS2R38 haplotypes, classifying participants as super-tasters, tasters, or non-tasters. Analysis of Covariance (ANCOVA) models were used to examine differences among the three TAS2R38 genetic groups in bitter taste sensitivity, food cravings, body mass index (BMI), non-fasting blood glucose, and family history of type 2 diabetes (T2D).

Results: A significant difference in sensitivity to the bitter taste compounds thiourea and phenylthiocarbamide (PTC) was observed among the three TAS2R38 genetic groups (*P* < 0.01). The sensitivity scores of the bitter taste compounds thiourea and PTC were lowest for super-tasters and highest for non-tasters (-41.283 and -0.233 for thiourea; -32.983 and 3.380 for PTC, respectively). Moreover, super-tasters had a significantly higher cravings score than non-tasters for starchy foods (2.641 vs. 2.183, respectively). Tasters had a significantly higher cravings score than non-tasters for bitter foods (2.306 vs. 1.740, respectively). No significant differences were observed across the TAS2R38 genetic-based taste groups for the chronic disease risk factors, BMI non-fasting blood glucose, and family history of T2D.

Conclusions: Super-tasters had the lowest taste sensitivity for bitter compounds and the highest cravings for bitter foods, which suggests that they might be more likely to consume bitter foods. Examining the genetic differences in taste preferences and sensitivities may pave the way for effective and individualized dietary plans and intervention strategies targeting chronic disease prevention, treatment, and management.

## Introduction

Nutrition-related diseases are highly prevalent in the United States, with more than 70% of adults being overweight/obese, 50% having diabetes/prediabetes, and 50% having cardiovascular disorders [1-3]. These chronic conditions have an adverse impact on the healthcare systems, the economy, and overall public health [1,4]. Yet, despite these dire repercussions, most Americans follow poor dietary patterns, do not meet federal dietary recommendations for all of the nutrient-rich food groups such as fruits, vegetables, and whole grains, and overconsume energy from solid fats, added sugars, and alcoholic beverages [5]. Therefore, it is imperative to comprehend the chief determinants of food preferences and food selection to understand nutritional behavior and prevent diet-related chronic diseases.

Food preference is complex and is influenced by the interactions between genetic factors, environmental determinants, and taste sensation [6-9]. Tastants, water-soluble chemicals in food, stimulate taste receptor cells and produce a taste sensation. The genotypes of these receptors, particularly their genetic single nucleotide polymorphisms (SNPs), lead to inter-individual variability in taste perception [10]. For instance, the ability to perceive bitter taste is highly variable among humans, which may be related to the genetic polymorphisms implicated in the taste receptor genes.

The stimuli that contribute to the perception of the bitter taste bind to members of the receptor protein family TAS2R [11]. The bitter taste receptor, type 2 member 38 (TAS2R38), coded by the gene TAS2R38, is one of the most widely studied bitter-taste genes, responsible for the perception of the bitter taste of compounds that contain the thiocyanate chemical group [12,13], and crucial for investigating the contribution of genetic variation to bitter-taste perception [14,15].

Three SNPs of the TAS2R38 gene lead to two common haplotypes, proline-alanine-valine (PAV) and alanine-valine-isoleucine (AVI) [14,16]. Based on their sensitivity to bitter-tasting compounds, individuals can be classified as super-tasters, tasters, or non-tasters [17], with tasters and super-tasters having one or two copies of the PAV haplotype, respectively, and non-tasters having the AVI haplotype [14,16]. Tasters and super-tasters perceive bitter taste more intensely and are thus more likely to avoid bitter-tasting foods such as cruciferous vegetables, which play a crucial role in reducing the risk of various chronic diseases [18].

Even though various sensory studies suggest that TAS2R38 haplotypes are accountable for the degree of bitterness perceived [19-21], there is not enough evidence in observational studies for the association between TAS2R38 haplotypes and food cravings, which are the main determinants of food intake. Furthermore, more research is required to understand the relationship between TAS2R38 haplotypes and various risk factors to be able to provide additional insights into disease prevention and management.

We have conducted a study to examine whether or not genetically defined taster groups would differ in their sensitivity to the bitter-tasting compounds thiourea and phenylthiocarbamide (PTC) and in cravings for various foods and food groups. We also examined whether or not there were significant differences between these groups in terms of chronic disease risk factors, including body mass index (BMI), non-fasting blood glucose, and family history.

## Materials and methods

Design and methods

In a cross-sectional study, 116 individuals (57 males, 59 females) were recruited from Loma Linda University (LLU) campus in California. To be eligible to participate in the study, individuals had to be non-diabetic, aged between 18 and 45 years, had consumed at least one bitter food in their lifetime, had a normal appetite, and not have had a history of ageusia, dysgeusia, food allergies, or eating disorders, as determined by the SCOFF questionnaire [22]. Each participant provided informed consent and completed the self-report surveys.

Participants took 20 to 25 minutes to complete all parts of the study and were given the option to enter a raffle for one of eight $25 gift cards. Only individuals who were interested in the gift card raffle were required to provide their email addresses. The study was approved by LLU’s Institutional Review Board (IRB# 5140380).

Measurements

Various measures were carried out to assess the participants' demographics, bitter sensitivity, anthropometrics, and food cravings.

Altered Food Craving Inventory

The Altered Food Craving Inventory (FCI-A) is a reliable and valid measure of general and specific food cravings and includes a 31-item survey derived from the original FCI that assesses the frequency of food cravings in the past month on a five-point scale (1 = never to 5 = always/almost every day) [23,24]. The FCI-A responses were averaged to yield an overall (aggregate) score and subscale scores, with higher numbers reflecting more frequent cravings for the food category.

Bitter Sensitivity Taste Test

Taste sensitivity was measured by rating the participants’ degree of liking or disliking of a concentrated solution or test strip containing the relevant chemical compound(s) on a general labeled magnitude scale (gLMS) [25,26]. To assess bitter taste sensitivity, participants were asked to taste three paper test strips from Indigo® Instruments (Waterloo, Ontario), with one being a control (neutral), one infused with thiourea, and one infused with PTC [27]. Taste sensitivity runs on a continuum, and responses categorize individuals into three groups: non-tasters, tasters, and super-tasters [28,29].

The presentation of the three taste test strips was counterbalanced to reduce false elicited responses [30]. Participants were required to rinse their mouths with bottled water provided by the researcher to cleanse their palates before and in between each test strip evaluation [31]. Then, the respondents used the validated gLMS to record how much they liked or disliked the test strip. The gLMS evaluation form included scores that ranged from -100 to +100 [25]. A constant was then added to eliminate negative values, resulting in values between 0 and 200 for thiourea and control and between 0 and 170 for PTC. Values closer to 200 or 170 indicated that the participants liked the taste of the test strip and were less sensitive to the bitter taste. Values closer to 0 indicated that the participants disliked the taste of the test strip. The gLMS values were normally distributed and did not require transformation.

Salivary Genetic Data Collection

The saliva was examined to assess and identify the participants' TAS2R38 bitter taste sensitivity gene and to group them according to genetically based taste sensitivity. First, participants were asked to rinse their mouths with bottled water provided by the researcher before saliva collection. Then, they were required to submit a 2ml saliva sample using the Oragene DNA Genotek kits (Ottawa, Ontario). The saliva was collected in a cylindrical container. Upon collecting the sample, ethyl alcohol was mixed in to preserve the samples, which were then sent to a genetic analysis service, GenoFIND®.

Genetic material (700 microL) was extracted to genotype three single nucleotide polymorphism (SNP) using the Taqman® allele assay. The three SNPs within the TAS2R38 gene analyzed were rs10246939, rs713598, and rs1726866, which gave rise to two common haplotypes, named PAV and AVI, respectively. Based on the sample, there were three possible pairs of TAS2R38 amino acid codon triplets arrangements: super-tasters (PAV/PAV), tasters (PAV/AVI), and non-tasters (AVI/AVI).

Blood Glucose

Each participant’s index finger was pricked to obtain a blood sample to assess non-fasting blood glucose using OneTouch Ultra Mini® (LifeScan, Inc., Wayne, PA). Non-fasting blood glucose (mg/dL) was collected on glucose test strips and read by a calibrated glucometer.

Anthropometric Measures

Weight and height were measured with a digital scale and standiometer, respectively. Weight (lb) and height (inches) were converted to kilograms and meters to calculate BMI with units of kg/m^2^. 

Demographic Survey

A demographic survey was administered to collect data on the study population's basic characteristics, including age, gender, race, occupation, education level, and marital status.

Statistical methods

According to the sample size calculations, using an effect size of 0.066 (coefficient of determination) for the correlation between bitter taste sensitivity and fat intake [32], and with alpha set at 0.05, the study required a minimum sample size of 115 participants to achieve at least 80% power.

Data were analyzed using SPSS version 25 (IBM Corp., Armonk, NY), with a level of significance set at α = 0.05.

Differences in the basic characteristics between the three genetically defined taster groups were assessed by one-way analysis of variance (ANOVA) or χ2 test, as appropriate. Data for quantitative variables were presented as mean ± standard deviation (SD), and data for qualitative variables were displayed as frequency (percentage).

Analysis of Covariance (ANCOVA) models were used to estimate significant differences across the three genetically defined taster groups in bitter taste sensitivity to thiourea and PTC, in various food cravings, and in two chronic disease risk factors (BMI and non-fasting blood glucose). The model was adjusted for age, gender, body weight, and race. To examine the correlation between the genetically defined taster groups and having a family history of type 2 diabetes (T2D), a χ2 test of independence was carried out. Several assumptions were satisfied before conducting the analysis. All the continuous variables were normally distributed and appropriate for data analysis.

## Results

Table 1** **summarizes and compares the basic characteristics of the three groups. A total of 116 individuals participated in the study. The mean ages of the study participants were 26.56, 27.71, and 28.00 for non-tasters, tasters, and super-tasters, respectively. The mean body weights were 162.26, 159.56, and 160.28 lbs for the three groups, respectively. The majority of participants were single (21, 67.7%; 34, 81%; and 32, 76.2%, for non-tasters, tasters, and super-tasters, respectively), were students (26, 83.87%; 27, 64.29%; and 34, 79.07%, respectively), and had attained a bachelor’s degree (13, 41.9%; 20, 47.6%; and 20, 46.5%, respectively). No significant differences were observed between the three groups for any basic characteristics, except for race; most participants were White (15, 48.4%; 18, 42.9%; and 12, 27.9% for non-tasters, tasters, and super-tasters, respectively; *P* = 0.033).

**Table 1:** Basic characteristics of the study population (n = 116). *P*-values are generated through χ2 for categorical variables and independent t-test for continuous variables. ^a^Data are displayed as frequency (percentage) for nominal/categorical variables and as mean ± standard deviation for scale variables. *Statistically significant *P*-value.

Variable	Non-taster	Taster	Super-taster	*P*-value
Gender				0.693
Male	13 (43.3%)	21 (50.0%)	23 (53.5%)	
Female	17 (56.7%)	21 (50.0%)	20 (46.5%)	
Race				0.033*
White	15 (48.4%)	18 (42.9%)	12 (27.9%)	
Asian	4 (12.9%)	16 (38.1%)	21 (48.8%)	
Black or African American	6 (19.4%)	1 (2.4%)	6 (14.0%)	
Native Hawaiian or other Pacific Islander	1 (3.2%)	3 (7.1%)	1 (2.3%)	
Decline to state	5 (16.1%)	4 (9.5%)	3 (7.0%)	
Marital status				0.463
Single	21 (67.7%)	34 (81.0%)	32 (76.2%)	
Married	10 (32.3%)	8 (19.0%)	9 (21.4%)	
Other	0 (0)	0 (0)	1 (2.4%)	
Occupation				0.565
Student	26 (83.87%)	27 (64.29%)	34 (79.07%)	
Other	5 (16.13%)	15 (35.71%)	9 (20.93%)	
Education				0.957
High School Diploma or GED	1 (3.2%)	1 (2.4%)	2 (4.7%)	
Some college	6 (19.4%)	4 (9.5%)	4 (9.3%)	
Associate degree	1 (3.2%)	2 (4.8%)	3 (7.0%)	
Bachelor’s degree	13 (41.9%)	20 (47.6%)	20 (46.5%)	
Master’s degree	5 (16.1%)	7 (16.7%)	9 (20.9%)	
Doctoral degree	5 (16.1%)	7 (16.7%)	5 (11.63%)	
Age (years)	26.560 ± 4.693	27.710 ± 5.334	28.000 ± 5.951	0.645
Weight (lbs.)	162.260 ± 36.791	159.56 ± 37.095	160.28 ± 36.293	0.625
Blood glucose (mg/dL)	130.890 ± 12.471	133.210 ± 16.689	129.380 ± 16.140	0.296
BMI (kg/m^2^)	25.080 ± 3.780	24.436 ± 4.103	25.241 ± 3.948	0.315

Tables 2-4 display the findings of the ANCOVA models. The group differences in bitter taste sensitivity among the three TAS2R38 genetically defined taster groups are shown in Table 2. The gLMS scores of the bitter taste compound thiourea were -0.233, -21.544, and - 41.283 among non-tasters, tasters, and super-tasters, respectively. The gLMS scores of the bitter taste compound PTC were 3.380, -19.629, and -32.983 among non-tasters, tasters, and super-tasters, respectively. There was a significant difference in sensitivity between all groups (*P* < 0.01). Post hoc analyses revealed significant differences between non-tasters and super-tasters for thiourea (-0.233 vs. - 41.283, respectively; 95% confidence interval [CI] for the difference in means 15.163-66.938) and PTC (3.380 vs. -32.983, respectively; 95% CI 11.104-61.623).

**Table 2:** Differences in bitter taste sensitivity among the three TAS2R38 genetic groups (n = 116). Data are expressed as means and 95% confidence interval (CI) after adjusting age, gender, body weight, and race. ^a^The *P*-value of the Analysis of Covariance (ANCOVA) model was used to assess the overall significant differences in taste sensitivity to two bitter compounds (thiourea and PTC) between the three TAS2R38 genetic groups. *P < 0.05.

	Non-taster		Taster		Super-taster		(Non-taster)-(Taster)		(Non-taster)-(Super-taster)		(Taster)-(Super-taster)		*P*^a^
	Mean	95% CI	Mean	95% CI	Mean	95% CI	Mean difference	95% CI	Mean difference	95% CI	Mean difference	95% CI	
Thiourea-gLMS	-0.233	-17.168 to 16.703	-21.544	-35.197 to -7.891	- 41.283	-53.658 to -28.908	21.311	-5.366 to 47.989	41.050	15.163-66.938	19.739	-2.909 to 42.386	0.001*
PTC-gLMS	3.380	-13.145 to 19.905	-19.629	-32.951 to -6.307	-32.983	-45.059 to -20.908	23.009	-3.021 to 49.040	36.364	11.104-61.623	13.354	-8.744 to 35.452	0.002*

**Table 3:** Differences in food cravings between the three TAS2R38 genetic groups (n = 116). Data are expressed as means and 95% confidence interval (CI) after adjusting for age, gender, body weight, and race. ^a^The *P*-value of the Analysis of Covariance (ANCOVA) model to assess the overall significant differences in food cravings scores between the three TAS2R38 genetic groups. *P < 0.05.

	Non-taster		Taster		Super-taster		(Non-taster)-(Taster)		(Non-taster)-(Super-taster)		(Taster)-(Super-taster)		*P*^a^
	Mean	95% CI	Mean	95% CI	Mean	95% CI	Mean Diff	95% CI	Mean Diff	95% CI	Mean Diff	95% CI	
FCI_Fat	1.828	1.428-2.221	2.115	1.795-2.435	2.190	1.900-2.480	-0.290	-0.915 to 0.335	-0.365	-0.972 to 0.241	-0.075	-0.606 to 0.455	0.264
FCI_Sweet	2.258	1.796 2.719	2.321	1.949-2.693	2.747	2.410-3.084	-0.064	-0.791 to 0.664	-0.489	-1.195 to 0.216	-0.426	-1.043 to 0.192	0.120
FCI_Starchy	2.183	1.785-2.582	2.409	2.088-2.730	2.641	2.350-2.932	-0.032	-0.854 to 0.402	-0.458	-1.067 to -0.273	-0.232	-0.765 to 0.301	0.039*
FCI_Bitter	1.740	1.360-2.116	2.306	2.003-2.609	2.027	1.752-2.302	-0.566	-1.159 to -0.027	-0.287	-0.862 to 0.289	0.279	-0.224 to 0.782	0.042*

**Table 4:** Differences in chronic disease risk factors between the three TAS2R38 genetic groups (n = 116). Data are expressed as means and 95% confidence intervals (CI) after adjusting for age, gender, and race. ^a^The *P*-value of Analysis of Covariance (ANCOVA) model to assess the overall significant differences in chronic disease risk factors between the three TAS2R38 genetic groups.

	Non-taster		Taster		Super-taster		(Non-taster)-(Taster)		(Non-taster) – (Super-taster)		(Taster)-(Super Taster)		*P*^a^
	Mean	95% CI	Mean	95% CI	Mean	95% CI	Mean difference	95% CI	Mean difference	95% CI	Mean difference	95% CI	
BMI	24.940	22.743-27.137	24.326	22.554-26.097	25.965	24.360-27.571	0.614	-2.847 to 4.076	-1.025	-4.384 to 2.334	-1.640	-4.578 to 1.299	0.935
Non-fasting blood glucose	130.316	121.861-138.771	132.916	125.740-140.092	135.273	128.992-141.554	-2.600	-16.235 to 11.034	-4.957	-17.965 to 8.051	-2.357	-14.065 to 9.351	0.933

As shown in Table 3, ANCOVA analyses revealed significant differences across the TAS2R38 genetic-based taste groups for starchy and bitter foods (*P*-values = 0.039 and 0.042, respectively). Super-tasters had a significantly higher cravings score for starchy foods than non-tasters (2.641 vs. 2.183, respectively; 95% CI for the difference in means -1.067 to -0.273). Tasters had a significantly higher cravings score for bitter foods than non-tasters (2.306 vs. 1.740, respectively; 95% CI for difference in means -1.159 to -0.027).

ANCOVA analyses revealed no significant differences across the TAS2R38 genetic-based taste groups for two diabetes risk factors, BMI and non-fasting blood glucose (*P*-values = 0.935 and 0.933, respectively) (Table 4)

As shown in Table 5, the chi-square test of independence revealed no significant correlation between a family history of T2D and TAS2R38 genetic groups (*P* = 0.364).

**Table 5:** Correlation between family history of type 2 diabetes (T2D) and the three TAS2R38 genetic groups (n = 116). Data are expressed as frequency (percentage). ^a^The *P*-value of the chi-square test of independence was used to assess the correlation between family history of T2D and TAS2R38 genetic groups.

		TAS2R38 genetic groups			*P*^a^
		Non-taster	Taster	Super-taster	
Family history of T2D	Yes	8 (25.8%)	8 (19.0%)	14 (32.6%)	0.364
	No	23 (74.2%)	34 (81.0%)	29 (67.4%)	

## Discussion

Our study showed that bitter taste sensitivity differs significantly between super-tasters, tasters, and non-tasters. The gLMS scores of the bitter taste compounds thiourea and PTC were lowest for super-tasters and highest for non-tasters. Non-tasters had a significantly lower cravings score for starchy and bitter foods than super-tasters and tasters. This suggests that super-tasters and tasters are more likely to consume bitter foods, including cruciferous vegetables and starchy foods, respectively, than non-tasters. We did not observe significant differences across the TAS2R38 genetic-based taste groups for other food cravings, BMI, or non-fasting blood glucose. There was no correlation between a family history of T2D and TAS2R38 genetic-based taste groups.

Contrary to our findings, meager evidence suggests that tasters and supertasters perceive bitter taste more intensely [14,33]. However, the link between TAS2R38 haplotypes and various bitter and sweet-taste outcomes is highly inconsistent [34-39]. There is a general lack of agreement in the literature when it comes to the relationship between TAS2R38 haplotypes and the consumption or preference for bitter-tasting foods in observational studies [35,40-48]. Moreover, findings from studies are not congruent when it comes to the association between TAS2R38 haplotypes and risk factors for various chronic diseases [49,50]. Meng and Nielsen [50] showed no significant difference in BMI between super-tasters, tasters, and non-tasters. The inconsistencies could be related to various methodologic and biological factors that have not been well-examined and controlled for [51].

The discrepancy between our findings and the literature could be related to our population. The study sample was composed mainly of individuals with a normal BMI, who were health-conscious, followed a healthy dietary pattern, and exercised regularly [52-54]. Thus, the environment at Loma Linda and the participants' healthy lifestyles could have been significant determinants of their health status, food preferences, and cravings that surpass the genetic polymorphism. The possible homogeneity in lifestyles between the three groups could have led to the lack of differences in disease risk factors and nonsignificant findings. The health consciousness among this population could have influenced their overall diet and craving for healthier bitter and starchy foods [55]. The higher cravings observed among tasters and super-tasters could be for healthier, starchy, and bitter foods such as whole grains, legumes, fruits and kale, Brussels sprouts, and other cruciferous vegetables.

To our knowledge, this was the first examination of bitter taste sensitivity and its association with food cravings and disease risk factors in a healthy population. We utilized validated surveys to examine bitter sensitivity and food cravings, which enhanced internal validity. The study, however, had several limitations. First, we did not delve into the participants’ dietary habits and thus did not adjust for dietary factors and total energy. Moreover, since we recruited from a school of public health and the university’s health and wellness center, most of the study participants were young and healthy. Our participants' lifestyle and dietary patterns may differ from others who do not practice the same healthy lifestyle. This might impact external validity since the study sample does not represent the larger population. In addition, since only non-diabetic participants between 18 and 45 years old were included in the investigation, we were unable to compare diabetic and non-diabetic individuals.

Future studies are recommended to collect dietary data with tools such as 24-hour dietary recall or three-diet records to understand better how nutritional patterns influence the association between TAS2R38 haplotypes and food cravings. Examining the link between TAS2R38 haplotypes and food cravings in vegetarians and non-vegetarians will provide additional insight into the role that diet plays in this association. Moreover, diabetic and non-diabetic individuals should be compared in terms of genetic variations to taste sensitivity and how this relates to bitter food cravings. Examining genetic differences in taste preferences and sensitivities may be an effective strategy for establishing more tailored approaches to dietary interventions for the prevention, treatment, and management of chronic diseases [56]. Non-tasters should be provided with dietary guidance to consume healthy starchy and bitter foods such as cruciferous vegetables to attenuate their chronic disease risks [18].

## Conclusions

Super-tasters and tasters, categorized based on the TAS2R38 haplotypes, were less sensitive to bitter taste compounds and had higher food cravings for starchy and for bitter foods than non-tasters. This indicates that super-tasters and tasters could be more likely to consume bitter foods and starchy foods, respectively, than non-tasters. No significant differences were observed across the TAS2R38 genetic-based taste groups for BMI, non-fasting blood glucose, and family history of T2D. This suggests that environmental and lifestyle factors have a stronger influence on food cravings and disease risk than genetic polymorphism. Future studies should examine dietary patterns to understand better how they influence the association between TAS2R38 haplotypes and food cravings. Exploring the genetic differences in taste preferences and sensitivities may pave the way for dietary intervention strategies that are more individualized and effective in the prevention, treatment, and management of chronic diseases.
